# Natural immunity to malaria preferentially targets the endothelial protein C receptor-binding regions of PfEMP1s

**DOI:** 10.1128/msphere.00451-23

**Published:** 2023-10-04

**Authors:** Madison A. Tewey, Drissa Coulibaly, Jonathan G. Lawton, Emily M. Stucke, Albert E. Zhou, Andrea A. Berry, Jason A. Bailey, Andrew Pike, Antoine Dara, Amed Ouattara, Kirsten E. Lyke, Olukemi Ifeonu, Matthew B. Laurens, Matthew Adams, Shannon Takala-Harrison, Amadou Niangaly, Bourema Kouriba, Abdoulaye K. Koné, J. Alexandra Rowe, Ogobara K. Doumbo, Jigar J. Patel, John C. Tan, Philip L. Felgner, Christopher V. Plowe, Mahamadou A. Thera, Mark A. Travassos

**Affiliations:** 1 Malaria Research Program, Center for Vaccine Development and Global Health, University of Maryland School of Medicine, Baltimore, Maryland, USA; 2 Malaria Research and Training Center, University of Sciences, Techniques and Technologies, Bamako, Mali; 3 Centre for Immunity, Infection and Evolution, Institute of Immunology and Infection Research, School of Biological Sciences, University of Edinburgh, Edinburgh, United Kingdom; 4 Roche NimbleGen, Inc., Madison, Wisconsin, USA; 5 Division of Infectious Diseases, Department of Medicine, University of California, Irvine, California, USA; University of Michigan, Ann Arbor, Michigan, USA

**Keywords:** Malaria, *Plasmodium falciparum*, PfEMP1, variant surface antigen, CIDRα1, endothelial protein C receptor, peptide, microarray, severe malaria

## Abstract

Antibody responses to variant surface antigens (VSAs) produced by the malaria parasite *Plasmodium falciparum* may contribute to age-related natural immunity to severe malaria. One VSA family, *P. falciparum* erythrocyte membrane protein-1 (PfEMP1), includes a subset of proteins that binds endothelial protein C receptor (EPCR) in human hosts and potentially disrupts the regulation of inflammatory responses, which may lead to the development of severe malaria. We probed peptide microarrays containing segments spanning five PfEMP1 EPCR-binding domain variants with sera from 10 Malian adults and 10 children to determine the differences between adult and pediatric immune responses. We defined serorecognized peptides and amino acid residues as those that elicited a significantly higher antibody response than malaria-naïve controls. We aimed to identify regions consistently serorecognized among adults but not among children across PfEMP1 variants, potentially indicating regions that drive the development of immunity to severe malaria. Adult sera consistently demonstrated broader and more intense serologic responses to constitutive PfEMP1 peptides than pediatric sera, including peptides in EPCR-binding domains. Both adults and children serorecognized a significantly higher proportion of EPCR-binding peptides than peptides that do not directly participate in receptor binding, indicating a preferential development of serologic responses at functional residues. Over the course of a single malaria transmission season, pediatric serological responses increased between the start and the peak of the season, but waned as the transmission season ended.

**IMPORTANCE:**

Severe malaria and death related to malaria disproportionately affect sub-Saharan children under 5 years of age, commonly manifesting as cerebral malaria and/or severe malarial anemia. In contrast, adults in malaria-endemic regions tend to experience asymptomatic or mild disease. Our findings indicate that natural immunity to malaria targets specific regions within the EPCR-binding domain, particularly peptides containing EPCR-binding residues. Epitopes containing these residues may be promising targets for vaccines or therapeutics directed against severe malaria. Our approach provides insight into the development of natural immunity to a binding target linked to severe malaria by characterizing an “adult-like” response as recognizing a proportion of epitopes within the PfEMP1 protein, particularly regions that mediate EPCR binding. This “adult-like” response likely requires multiple years of malaria exposure, as increases in pediatric serologic response over a single malaria transmission season do not appear significant.

## INTRODUCTION

Of the 625,000 and 619,000 deaths caused by malaria in 2020 and 2021, respectively, 76% were children under 5 years old, primarily in sub-Saharan Africa (1). The 2020 peak represents 57,000 more deaths than in 2019, a 10% increase in mortality that has been attributed to disruptions due to the COVID-19 pandemic (1). Malaria is a life-threatening infectious disease caused by parasites in the genus *Plasmodium*, with the species *Plasmodium falciparum* primarily responsible for severe malaria cases (1). To reduce the deadly consequences of malaria infection, it may be helpful to identify antigens that could serve as potential severe malaria vaccine targets.

Variant surface antigens (VSAs) play a critical role in *P. falciparum* virulence by enabling adhesion to host cell receptors, leading to parasite sequestration in the vasculature (2). *P. falciparum* erythrocyte membrane protein-1s (PfEMP1s) (3, 4) are the best studied VSA family. The genes encoding PfEMP1s consist of two exons: the extracellular exon 1, consisting of cysteine-rich interdomain regions (CIDRs) and Duffy binding-like (DBL) domains that mediate host receptor binding, and the intracellular exon 2, which includes the acidic terminal sequence (ATS) that is relatively conserved across many PfEMP1 variants (5, 6). PfEMP1s include a subset of variants that binds to endothelial protein C receptor (EPCR) through CIDRα1 domains and whose expression is associated with severe malaria (7). Activated protein C binding to EPCR plays a role in regulating the inflammatory response (8), and binding of the EPCR receptor may result in host dysregulation of inflammation and anticoagulation, both pathophysiologic characteristics of severe malaria (7). The PfEMP1 family also includes variants that bind to the CD36 receptor, a scavenger receptor that plays a role in fatty acid uptake, phagocytosis, and angiogenesis (9). Variants that bind the CD36 receptor account for approximately 84% of PfEMP1s in the 3D7 reference genome (10). CIDRα2–6 domains are able to bind to the CD36 receptor, and proteins that bind this receptor are typically associated with uncomplicated rather than severe malaria (11).

Located on the infected erythrocyte surface during the blood stage of malaria, VSAs are targets of the human immune response. *P. falciparum* displays great antigenic diversity, evading host immune response and resulting in the gradual development of immunity in malaria-endemic regions through repeated exposure over multiple years (12). Opposing forces are likely at play in PfEMP1 sequence diversity: enough sequence conservation must be present to ensure binding to the desired host receptor, but too much conservation may lead to host immune recognition and removal.

Severe malaria, including cerebral malaria, is rare after age 5 in regions of high malaria transmission (13), possibly due in part to the acquisition of VSA antibodies over time. Acquisition of immunoglobin G (IgG) that binds PfEMP1 has been associated with immunity to severe malaria, and models predicting malaria immunity after only one or two severe episodes provide hope that a vaccine targeting PfEMP1 might confer protection to severe malaria (14, 15). The rate at which children in malaria-endemic regions develop natural immunity is poorly characterized, and scrutiny of serologic responses to surface antigens such as PfEMP1s may inform our understanding of how natural immunity develops over a single malaria transmission season.

The EPCR binding region of PfEMP1 proteins is within the CIDRα1 domain, with variants that mimic the structure of the natural EPCR ligand (7). Nine functional residues have been identified as the amino acids directly involved with EPCR-binding: four residues insert into the EPCR hydrophobic groove, and five residues provide stabilizing hydrogen bonds (16). An increased level of EPCR-binding PfEMP1s is correlated with severity of disease presentation (17), and antibodies against EPCR-binding PfEMP1 variants are associated with less severe disease presentation (18), indicating a direct link between PfEMP1 binding to EPCR and severe malaria pathology. There is high structural diversity within the EPCR-binding functional region (16). Only 14 of the approximately 220 amino acid residues in the domain are absolutely conserved across previously studied CIDRα1 variants, and only 22 residues are conserved in more than 90% of domain variants (16). The majority of these conserved residues are within the internal core of PfEMP1, likely stabilizing the structure and providing correct positioning for functional residue binding. In contrast, CIDRα1 surface residues display a greater diversity, including those in the EPCR-binding region. Residues at these functional locations vary widely, and CIDRα1 variants display a wide variety of EPCR-binding affinities. Antibody responses to these functional residues have not previously been examined. Natural immunity to uncomplicated malaria appears to develop more slowly than immunity to severe malaria (14). The comparison of serologic responses to the CIDRα1 versus CIDRα2–6 domains may uncover differences in how immunity to severe and uncomplicated malaria develops.

The goal of this study was to identify differences between adult and pediatric antibody responses to five PfEMP1 CIDRα1 domains to investigate a potential mechanism of age-related immunity to severe malaria. First, we aimed to determine the regions within this domain serorecognized by adult and pediatric antibodies. Second, we compared serologic responses of adults and children to PfEMP1 variants associated with severe or uncomplicated malaria by comparing a CIDRα1-containing variant, a CIDRα2.8-containing variant, and their associated ATS domains. Third, we sought to determine how pediatric serologic responses change over a single malaria transmission season. We hypothesized that these approaches would help characterize an “adult-like” serologic response to pathogenic malaria antigens and could inform how natural immunity to severe malaria develops.

## MATERIALS AND METHODS

### Study setting

The serum samples for this study were collected in Bandiagara, a Sahelian town in the Dogon region of east-central Mali. At the time of sample collection, Bandiagara consisted of ~14,000 inhabitants, which has grown to ~26,000 according to the 2009 census. *P. falciparum* infections account for 97% of malaria infections in this region, with the remaining 3% representing *P. malariae* and rare *P. ovale* infections. The frequency of malaria infections displays a highly seasonal pattern. Transmission is at its nadir in the middle of the dry season in March. The transmission season begins in June and ends in December. Transmission peaks at the height of the rainy season in September, with up to 60 *P*. *falciparum*-infected mosquito bites per person per month at the time of sample acquisition (19). Children in Bandiagara typically experience at least one clinical malaria episode per year (20).

### Study population

Peptide microarrays were probed with sera from Malian adults aged 18–55 (median age: 25) randomly selected from the control arm of a phase I AMA1 vaccine (FMP2.1/AS02A) trial (*n* = 10) (21) and Malian children aged 1 to 5 years (median age: 3.5 years) enrolled in the control arm of a phase II AMA1 vaccine (FMP2.1/AS02A) trial (*n* = 10) (22) (Table S1). Control volunteers did not receive a vaccine affecting their risk of malaria infection, allowing for observation of natural immunity. Adult serum samples were collected at the start of the malaria transmission season in June 2004 (21), and no participating adults had a febrile malaria episode during the study period, which included the transmission season (23). Pediatric serum samples were collected at three time points: the beginning of the transmission season in June 2007 (Day 0), the peak of transmission season in September 2007 (Day 90), and in the middle of the following dry season in February 2008 (Day 240) (22). Pediatric samples were randomly selected from the subset of participants who experienced at least one clinical malaria episode during the course of the study, defined as exhibiting malaria symptoms and parasitemia on a blood smear (23). Children were followed through both monthly active surveillance and also passive surveillance for malaria-like symptoms at sick visits to the study clinic (21, 22). No child experienced a severe malaria episode. Sera from malaria-naïve North American blood donors (*n* = 5) served as negative controls.

### Microarray design

The peptide microarray included a tiling design to represent the PfEMP1 variants with high affinities for EPCR binding [PfDd2_040005100 (CIDRα1.4), PF3D7_0425800 (CIDRα1.6a), PF3D7_0600200 (CIDRα1.8a), and PF3D7_1150400 (CIDRα1.4)] as well as PF3D7_0533100, a PfEMP1 variant containing the CIDRα1.3 domain subtype that does not bind EPCR (16). PF3D7_0100100 (CIDRα2.8) was selected as a representative variant for CD36-binding CIDRα domains (24). We generated a multiple sequence alignment of the six CIDRa1 amino acid sequences using MUSCLE (25) with default parameters and visualized the alignment using Jalview (26) (Fig. S1). This design incorporated linear peptides 16 amino acids in length, overlapping by 12 amino acids. Peptide microarrays were synthesized through light-directed solid-phase peptide synthesis, as previously described (27). Bound antibodies from sera samples were labeled with a fluorescent Alexa Fluor 647 anti-human IgG secondary antibody for spectroscopic quantification.

For PF3D7_0533100 and for severe malaria-associated EPCR-binding variants PfDd2_040005100, PF3D7_0533100, PF3D7_0425800, PF3D7_0600200, and PF3D7_1150400, peptides spanning the length of the CIDRα1 domain (e.g., peptides 481–733 in PF3D7_0425800) were analyzed in this study. Domains ranged from 64 to 66 peptides in length. Serologic responses to the highly conserved ATS domain may be indicative of the intensity of previous malaria exposure. We analyzed peptides spanning the length of the ATS domains as well as the CIDRα1 and CIDRα2.8 domains from PfDd2_040005100 (a high-affinity EPCR-binding variant) and PF3D7_0100100 (an uncomplicated malaria-associated CD36-binding variant), respectively.

As the functional binding residues within the CIDRα1 domain may be important in the development of immunity to severe malaria, peptides that contained two or more of these EPCR-binding residues were considered “potential binding peptides” and serologic responses against these peptides in adults and children were compared.

### Statistical analysis

#### Group serologic responses

Fluorescence intensity represented a measure of peptide serologic response. We analyzed two aspects of serologic response: serorecognition and seroreactivity. While there is overlap in amino acid sequence between neighboring peptides, each peptide was treated independently in this analysis, as has been done previously (28).

“Serorecognition” indicates the presence of a significant serologic response to a given peptide. Group serorecognition of a peptide was defined as a fluorescence intensity significantly higher than malaria-naïve North American controls, based on a Wilcoxon rank-sum test. We used McNemar’s test to determine whether sets of peptides recognized by adult sera were significantly different from those recognized by pediatric sera.

“Seroreactivity” is a quantitative measure of the magnitude of an antibody response to a given peptide, indicated on the array by the fluorescence intensity at a given peptide. We used a Wilcoxon rank-sum test to compare the seroreactivity of pediatric sera and adult sera to each peptide and identify peptides that display differential seroreactivity between the two groups.

#### Individual serologic responses

A serorecognized peptide for an individual was defined as a peptide against which the fluorescence intensity for an individual was at least two standard deviations higher than the average fluorescence intensity of the malaria-naïve North American controls at that peptide. We analyzed the proportion of serorecognized peptides within each domain for each individual adult or pediatric serum sample.

Subsequently, a Wilcoxon rank-sum test was used to compare adult versus pediatric serorecognized peptide proportions for each PfEMP1 domain analyzed, and to compare serorecognized peptide proportions between PfEMP1 domains within either the adults or pediatric age group (e.g., comparing the CIDRα1 domain of PfDd2_040005100 to the CIDRα1 domain of PF3D7_0100100 for adults). We used a paired Wilcoxon signed-rank test to compare serorecognized peptide proportions between two regions on the same PfEMP1 variant within each age group.

#### Changes in pediatric serologic response during the transmission season

For serorecognition, a Wilcoxon rank-sum test was used to identify peptides against which pediatric fluorescence intensity as a group was significantly higher than malaria-naïve North American controls at each time point, indicating a serorecognized peptide. For seroreactivity, a Wilcoxon signed-rank test was used to identify peptides against which seroreactivity significantly changed across time points by comparing the fluorescence intensities recorded at Days 0–90, Days 90–240, and Days 0–240.

## RESULTS

### Group serologic responses

#### Malian adult sera exhibited a broader and more intense response to peptides within the CIDRα1 domain than pediatric sera

**Fig 1:**
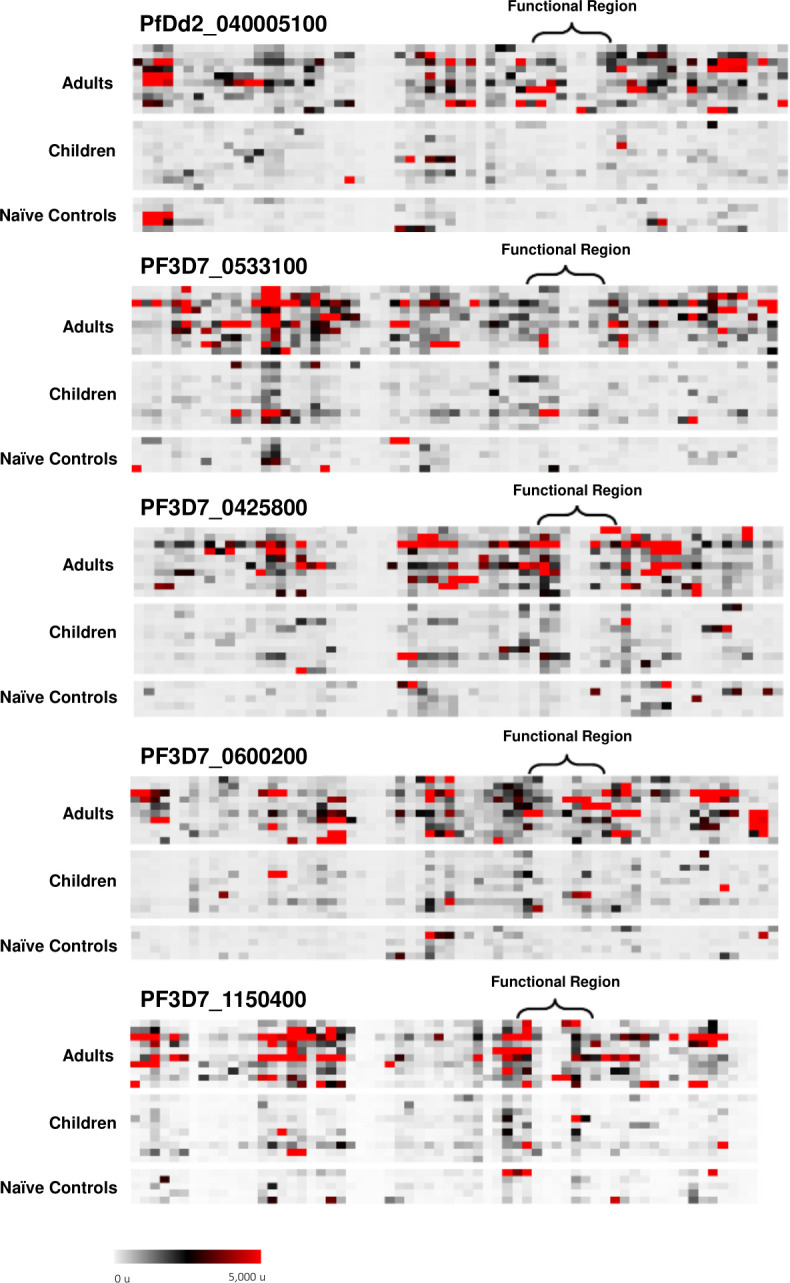
Seroreactivity heat maps of Malian adults and children against CIDRα1 domains. Fluorescence intensity of individual responses from Malian adult sera (*n* = 10), Malian pediatric sera (*n* = 10), and malaria-naïve North American sera (*n* = 5) to each peptide within the CIDRα1 domains of PfDd2_040005100, PF3D7_0533100, PF3D7_0425800, PF3D7_0600200, and PF3D7_1150400 are indicated. Each row represents an individual participant, and each column represents a peptide. High fluorescence intensity is indicative of high antibody response at a particular peptide. Participants are organized within groups by descending average fluorescence intensity.

**Fig 2:**
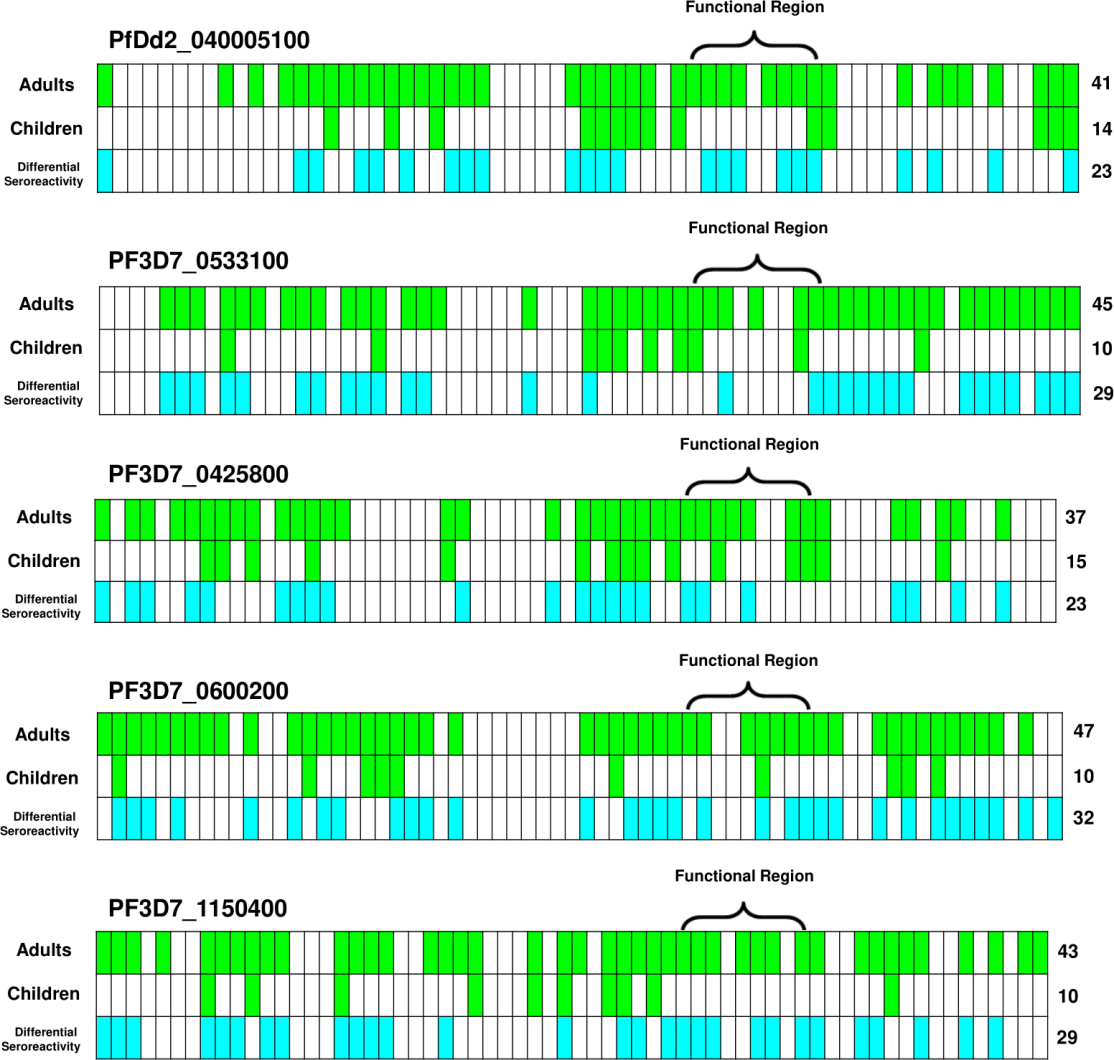
Identification of serorecognized and differentially seroreactive peptides. For PfEMP1 variants PfDd2_040005100, PF3D7_0533100, PF3D7_0425800, , PF3D7_0600200, and PF3D7_1150400, we identified significantly serorecognized peptides. Mean fluorescence intensity for each peptide was compared between either Malian adults (*n* = 10) or Malian children (*n* = 10) and the malaria naïve controls (*n* = 5) using a Wilcoxon rank-sum test (*α* = 0.05). Peptides that elicited a significantly higher response than controls are highlighted in green, indicating a serorecognized peptide. We also identified peptides with a significantly differential seroreactivity between Malian adult sera (*n* = 10) and Malian pediatric sera (*n* = 10) using a Wilcoxon rank-sum test (α = 0.05), highlighted in blue. The numbers on the right indicate the number of peptides against which there were significant serorecognition responses for the demographic (green) or the number of peptides against which the adult seroreactivity was significantly higher than the pediatric seroreactivity (blue).

Malian adults as a group serorecognized on average 70% of the CIDRα1 peptides on the peptide microarray, whereas Malian pediatric sera recognized on average only 20% of these peptides (Fig. 1 and 2). Without exception, all peptides recognized by pediatric sera were recognized by adult sera (Fig. 2).

Adult antibody seroreactivity was consistently higher than pediatric antibody response (Fig. 2). Adult sera reacted more intensely than pediatric sera to all peptides observed, with significantly differential responses for 42% of peptides observed (Fig. 2).

#### As a group, adult sera tended to recognize most potential binding peptides, whereas pediatric serorecognition was minimal

Of the nine potential binding peptides within each CIDRα1 domain variant, Malian adult sera recognized 7.0 peptides on average, whereas Malian pediatric sera recognized 1.4 peptides on average, indicating that serorecognition patterns were significantly different between adult and pediatric sera according to a McNemar’s test (*P* < 0.00001).

### Individual serologic responses

#### Malian adult sera consistently recognized a higher median percentage of peptides within each CIDRα1 domain variant than did Malian pediatric sera

**Fig 3:**
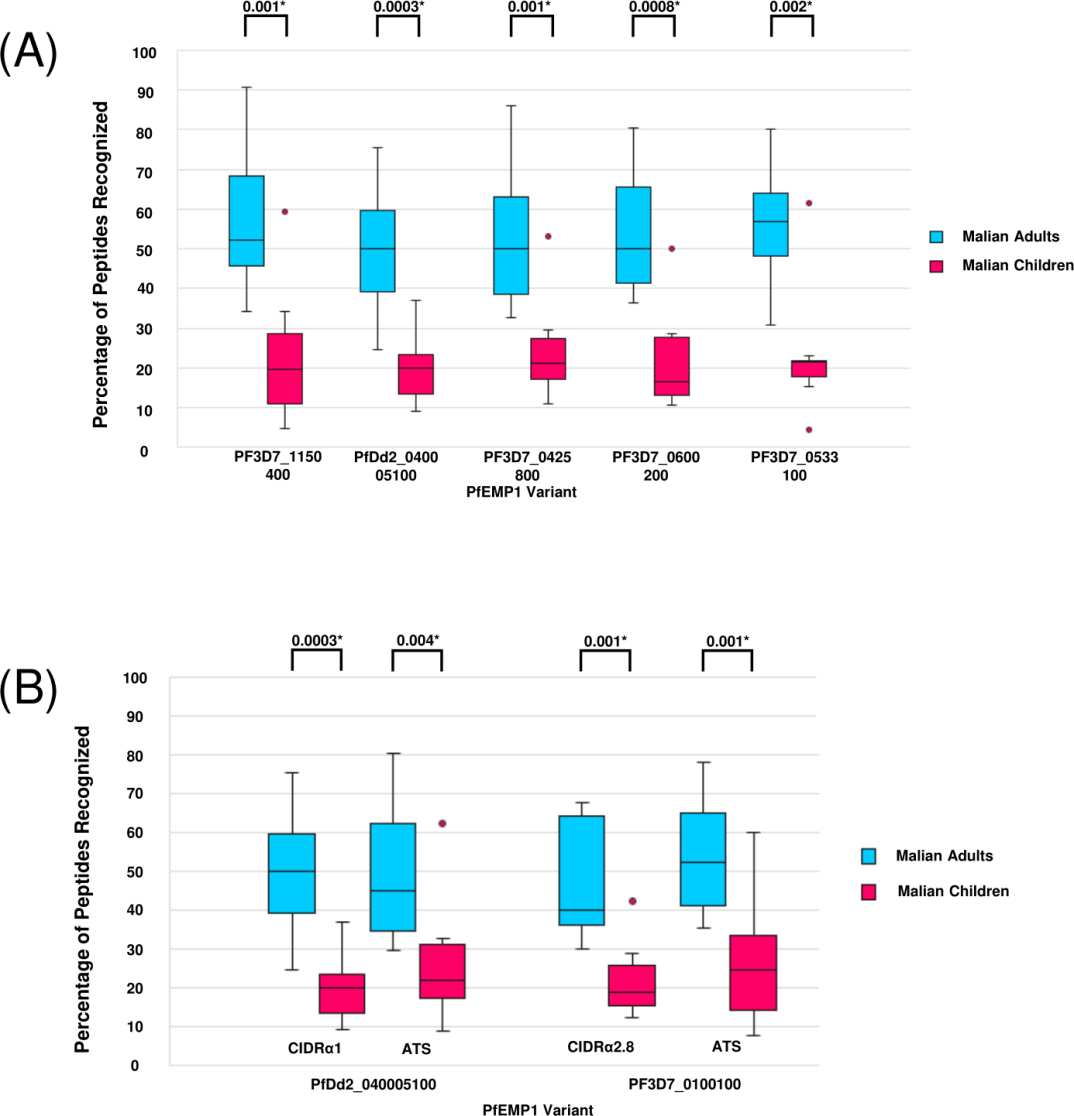
Proportion of serorecognized peptides among PfEMP1 CIDRα1 domain variants and within the receptor binding domains and ATS domains of PfDd2_040005100 and PF3D7_0100100. The percentage of serorecognized peptides within (**A**) each CIDRα1 domain variant recognized and (**B**) each binding domain variant (CIDRα1 of PfDd2_040005100 and CIDRα2.8 of PF3D7_0100100) and ATS region by Malian adults (*n* = 10) and Malian children (*n* = 10) are displayed. If fluorescence intensity at a peptide was at least two standard deviations above the malaria-naïve control average for a given participant, then this was considered a serorecognition response against that peptide. Each box plot shows the median, bracketed by the lower 25th and upper 75th percentiles, and the minimum and maximum values with outliers (proportional values 2.5 standard deviations greater than the median serorecognition proportion) indicated by dots. Significant serorecognition proportion differences according to a Wilcoxon rank-sum test (α = 0.05) are indicated by an asterisk.

Malian adult sera exhibited median peptide serorecognition percentages ranging between 50.0% and 56.9% for each of the five CIDRα1 domain variants. Malian pediatric sera exhibited a less robust response, with median peptide serorecognition percentages between 16.7% and 21.5% (Fig. 3A). One outlier was a child whose serologic responses consistently fell within the adult interquartile range of each respective CIDRα1 variant, suggesting that this child (age 4) had a more mature antibody response than the other children.

#### The proportion of serorecognized EPCR-binding peptides was typically higher than that for corresponding non-binding peptides

**Fig 4:**
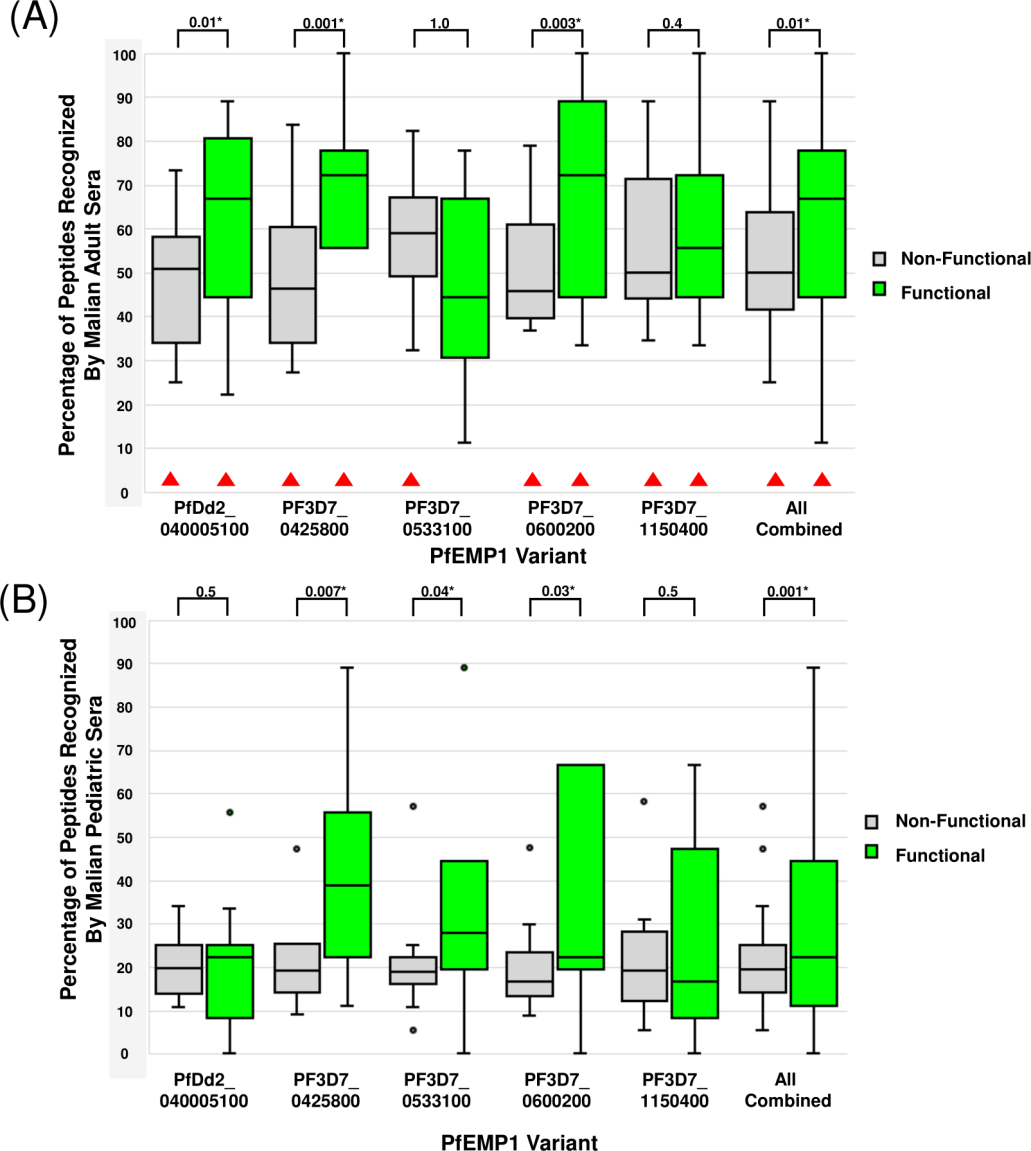
Proportion of serorecognized peptides among the non-binding versus potential binding regions of PfEMP1 CIDRα1 domain variants. The percentage of serorecognized peptides for (**A**) non-binding versus potential binding peptides for five PfEMP1 variants in Malian adults and (**B**) non-binding versus potential binding peptides of five PfEMP1 variants in Malian children. Brackets indicate *P* values for paired comparisons, with an asterisk (*) indicating a value significant at *α* = 0.05. In (**A**), red triangles indicate adult serorecognition proportions significantly higher than their pediatric counterparts (see Table S2 for *P* values). Each box plot shows the median, bracketed by the lower 25th and upper 75th percentiles, and the minimum and maximum values. Outliers, defined as proportion values 2.5 standard deviations above the serorecognition proportion median, are indicated by dots.

The proportion of potential binding peptides serorecognized was significantly higher than the proportion of non-binding peptides serorecognized in the CIDRα1 domain for both Malian adult sera (*P* < 0.01) and for Malian pediatric sera (*P* < 0.001) (Fig. 4; Wilcoxon signed-rank test). This was true for three of the five EPCR-binding variants for adult and for pediatric sera, and the largest gap in recognized peptide proportions differences was for PF3D7_0425800.

#### Serorecognized proportions of both EPCR-binding peptides and non-binding peptides were typically higher for Malian adults than children

Malian adults serorecognized a significantly higher proportion of both potential binding and non-binding peptides than Malian children for all five PfEMP1 variants analyzed, with the exception of PF3D7_0533100 potential binding peptides (*P* = 0.1) (Fig. 4; Table S2).

#### For Malian adult sera, the broad response to the ATS domain was similar to that for an EPCR-binding CIDRα1 domain, but not for a CD36-binding CIDRα domain

**Fig 5:**
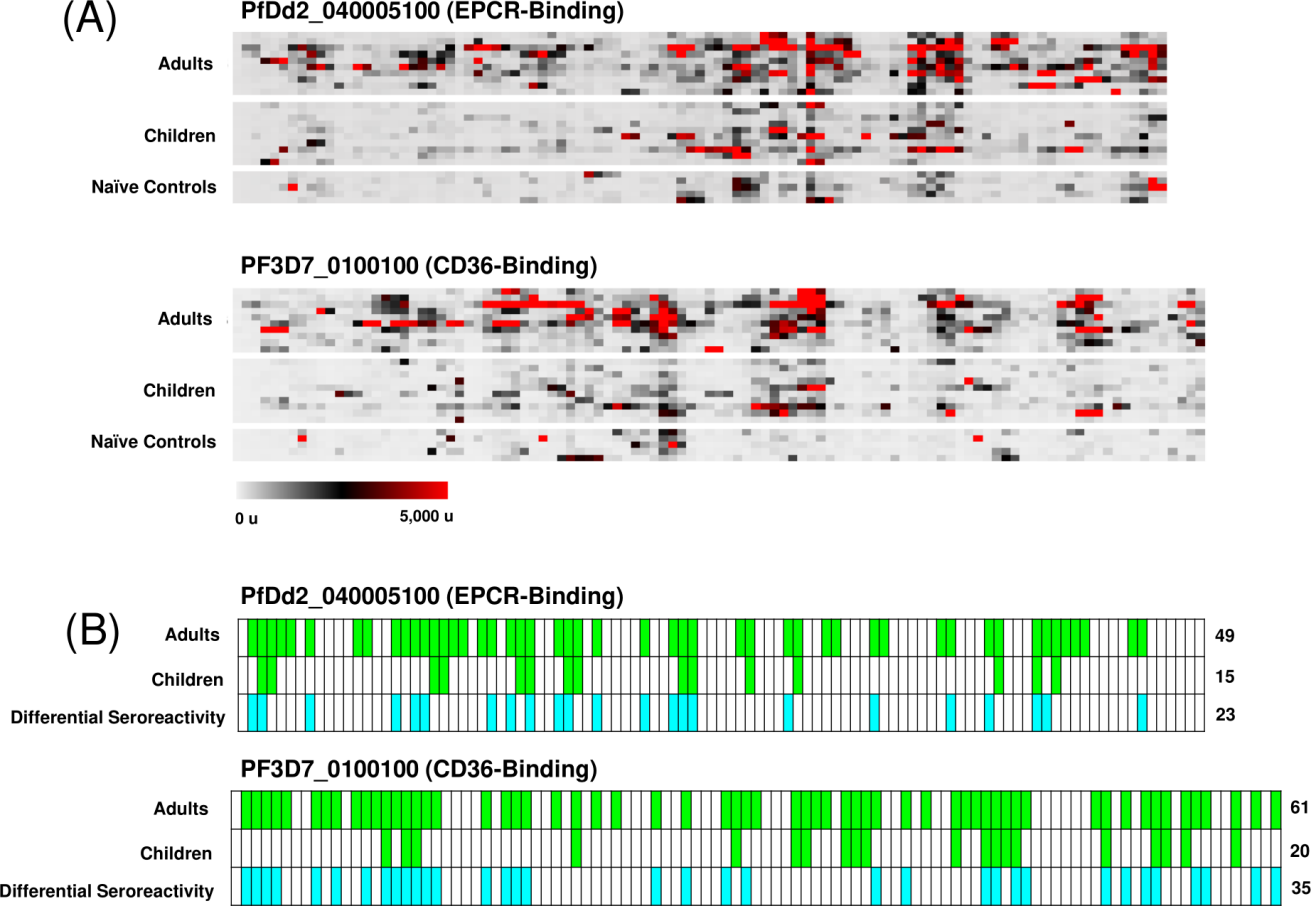
Seroreactivity heat maps of Malian adults and children and identification of serorecognized peptides within the ATS region of a severe malaria-associated and an uncomplicated malaria-associated PfEMP1 variant. (**A**) Fluorescence intensity of individual responses from Malian adult sera (*n* = 10), Malian child sera (*n* = 10), and malaria-naïve North American sera (*n* = 5) to each peptide within the ATS domains of (**A**) PfDd2_040005100 and (**B**) PF3D7_0100100 are indicated. Each row represents an individual participant, and each column represents a peptide. (**B**) For the severe malaria-associated PfEMP1 variant PfDd2_040005100 and uncomplicated malaria-associated PfEMP1 variant PF3D7_0100100, we identified significantly serorecognized peptides. Mean fluorescence intensity for each peptide was compared between either Malian adults (*n* = 10) or Malian children (*n* = 10) and the malaria naïve controls (*n* = 5) using a Wilcoxon rank-sum test. Peptides that elicited a significantly higher response than controls are highlighted in green, indicating a serorecognized peptide. We also identified peptides with a significantly differential seroreactivity between Malian adult sera (*n* = 10) and Malian pediatric sera (*n* = 10) using a Wilcoxon rank-sum test, highlighted in blue. The numbers on the right indicate the number of peptides against which there were significant serorecognition responses for the demographic (green) or the number of peptides against which the adult seroreactivity was significantly higher than the pediatric seroreactivity (blue).

Malian adult sera recognized a significantly higher proportion of peptides than Malian pediatric sera for the ATS regions of both the EPCR-binding PfEMP1 PfDd2_040005100 (*P* < 0.01) and the CD36-binding PfEMP1 PF3D7_0100100 (*P* < 0.01) (Fig. 5). There was no significant difference between the proportion of peptides recognized within the ATS of regions of PfDd2_040005100 and PF3D7_0100100 for Malian adult sera (*P* = 0.38) or for Malian pediatric sera (*P* = 0.79).

Interestingly, Malian adult sera recognized a similar proportion of peptides for the ATS region and CIDRα1 domain of PfDd2_040005100 (*P* = 1.0) but had higher proportions of serorecognized peptides for the ATS region of PF3D7_0100100 (*P* < 0.05) than for the corresponding CIDRα2.8 domain (Fig. 3B). Malian pediatric sera did not display significant differences in the proportions of peptides recognized between the CIDRα1 and ATS domains for PfDd2_040005100 (*P* = 0.13) or PF3D7_0100100 (*P* = 0.20).

#### Adult serorecognition of a CD36-binding CIDRα domain variant was significantly higher than pediatric serorecognition, reflecting the pattern observed in an EPCR-binding variant

Malian adult sera had a higher proportion of recognized peptides than Malian pediatric sera for both the CIDRα1 domain of PfDd2_040005100 (*P* < 0.001) and CIDRα2.8 domain of PF3D7_0100100 (*P* < 0.001). There was no significant difference in serorecognition percentage between the CIDRα1 and CIDRα2.8 domains for Malian adults (*P* = 0.38) or for Malian children (*P* = 0.849). When comparing mean fluorescence intensity for each participant for this CIDRα1 domain and CIDRα2.8 domain, both adult sera (*P* = 1.0) and pediatric sera (*P* = 0.1) had a similar seroreactivity for the two domains (Fig. S2).

### Changes in pediatric serologic response during the transmission season

#### Serologic responses to CIDRα1 peptides rose at the peak of the malaria transmission season, then declined as transmission waned

**Fig 6:**
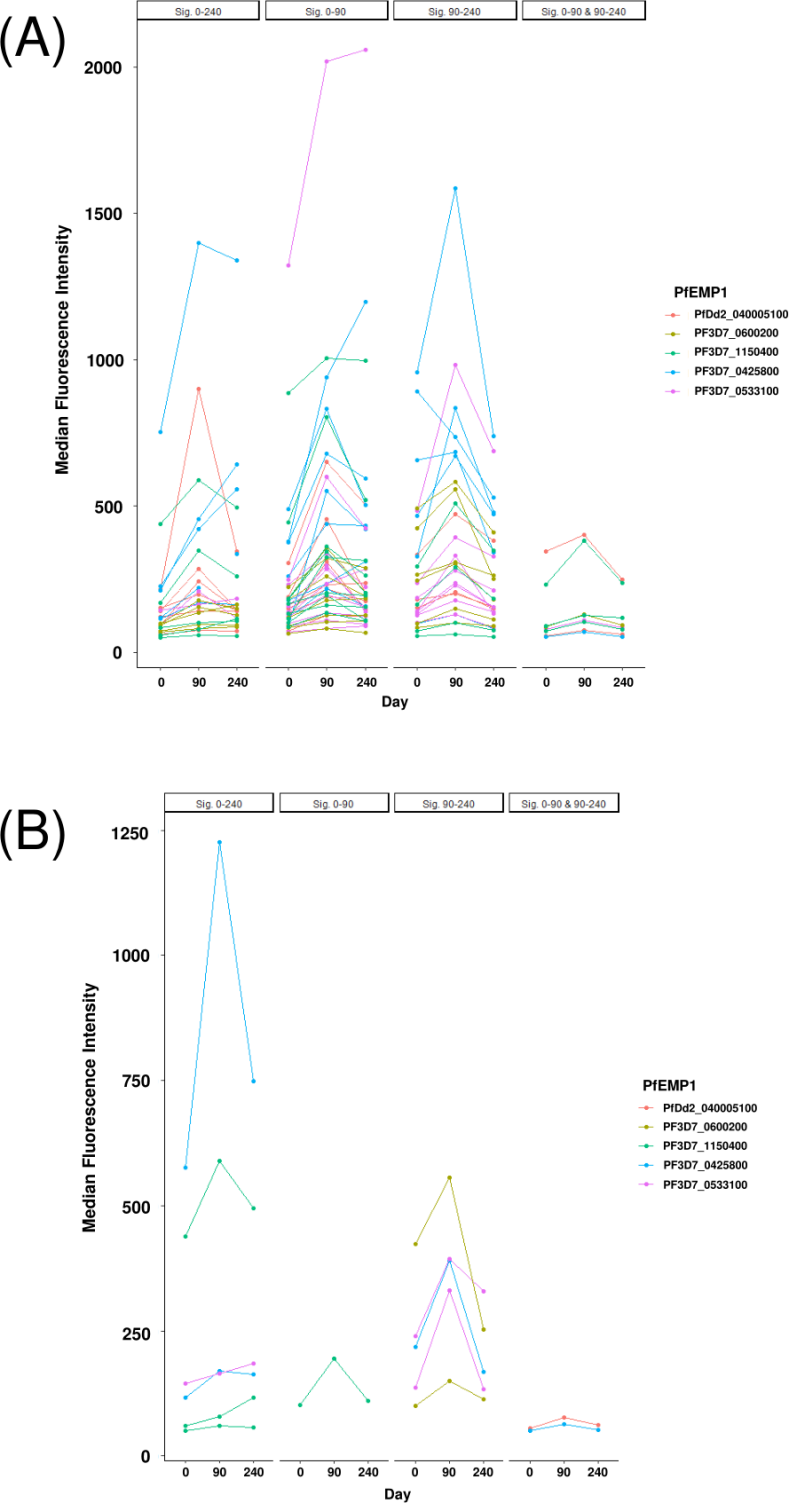
Changes in Malian pediatric seroreactivity over a single malaria transmission season. A paired Wilcoxon signed-rank test was used to compare the fluorescent intensities throughout the malaria transmission season at (**A**) every peptide or (**B**) potential binding peptides within the CIDRα1 domains of five PfEMP1 variants. A higher fluorescence intensity corresponds with a higher serologic response at a given peptide by the Malian pediatric cohort (*N* = 10). Each column depicts peptides within the CIDRα1 domain that had a significant change in average fluorescence intensity between the indicated time points. Day 0 represents the beginning of the malaria transmission season, Day 90 represents the height of the malaria transmission season, and Day 240 represents the offseason after transmission slows considerably.

Pediatric serologic response to peptides rose at peak malaria transmission season, then declined in the dry season (Fig. 6; Fig. S3 through Fig. S7). From Days 0 to 90, serologic responses to a subset of peptides in each PfEMP1 variant rose significantly, with a median proportion of 18.8% of peptides per variant (*n* = 5). From Days 90 to 240, there was a decline in serologic response to a subset of peptides for each variant, with a median proportion of 10.9% for such peptides (*n* = 5). Additionally, serologic responses increased significantly from Days 0 to 240 for a small subset of CIDRα1 peptides. Twenty-three of the 346 peptides (6.65%) elicited a significant increase (*α* = 0.05) in seroreactivity from Days 0 to 240, consisting of 6 of the 45 potential binding peptides (13.33%) and 17 of the 301 (5.65%) non-binding peptides (Fig. 6). The proportions of potential binding and non-binding domains with Days 0–240 increase were similar (*P* = 0.44; Wilcoxon signed-rank test).

## DISCUSSION

In this study, Malian adult sera had a more widespread and intense antibody response specific to the EPCR binding domain of multiple PfEMP1 variants than pediatric sera, and adult sera recognized all peptides recognized by pediatric sera. The EPCR receptor plays a crucial role in activating protein C, a homeostatic blood protease, associated with regulating the anticoagulant and anti-inflammatory pathways (29). Binding of PfEMP1 variants to EPCR could disrupt these pathways, potentially contributing to dysregulation and subsequent hyperinflammatory symptoms associated with severe malaria (30). Antibody responses to EPCR-binding PfEMP1s may yield insights into the development of naturally acquired immunity to severe malaria. As previously observed (15), serologic responses in this study display a pattern consistent with age-related antibody accumulation in geographic regions where individuals experience repeated malaria exposure. Age-related serologic responses therefore result from a combination of wider serorecognition and more intense reactivity at recognized regions in adult sera than pediatric sera. Our results suggest that a requisite level of antibody response across the EPCR-binding domain may be necessary for the recognition of EPCR-binding regions in particular and potentially for the development of natural immunity to severe malaria.

We have previously found that strong serologic responses to particular PfEMP1s are associated with milder clinical episodes of malaria (31). This may be due in part to protection against dysregulation of inflammatory pathways associated with PfEMP1-EPCR binding. We hypothesized here that antibody responses to the binding residues of CIDRα1 (defined as potential binding peptides) could play an important role in blocking PfEMP1 binding to the EPCR receptor. We expected that sera from malaria-exposed adults would have a particularly strong antibody response to the potential binding peptides of the CIDRα1 variants that bind EPCR. We found that both adults and children serorecognized a significantly higher proportion of EPCR-binding peptides than peptides that do not directly participate in receptor binding, indicating a preferential development of serologic responses at these functional residues. Epitopes containing these EPCR-binding residues may be promising targets for vaccines or therapeutics directed against severe malaria. Our findings suggest that these differential serologic responses begin in early childhood.

Interestingly, differences in serologic responses between adult and pediatric samples to potential binding and non-binding region peptides were most pronounced for the PfEMP1 variant PF3D7_0425800, which features a CIDRα1 domain that binds EPCR followed by a DBL domain that binds the intracellular adhesion molecule 1 (ICAM-1) receptor (32 – 34). This dual-binding phenotype may lead to the development of cerebral malaria through increased parasite sequestration and receptor signaling disruption (34), and further studies are warranted to determine if peptide serorecognition of this variant results in improved clinical outcomes. Our results additionally indicate that peptides of PF3D7_0533100, a VAR 1 PfEMP1 variant that does not bind EPCR and is sometimes classified as a pseudogene (7, 16), are bound by both adult and pediatric sera in similar proportions to variants known to be expressed, possibly indicating this protein is not a pseudogene or is closely related to a protein that is expressed on the surface of infected erythrocytes.

The sequence of the ATS domain is relatively conserved compared to other PfEMP1 domains (35) and therefore is thought to serve as an indicator of level of malaria exposure (36). Malian adult sera exhibited significantly higher levels of recognition and reactivity than pediatric sera to peptides within the two ATS domain variants analyzed. The proportion of serorecognized peptides in the ATS domains was similar to the peptide proportions recognized in the corresponding CIDR domains. However, a notable exception to this pattern was the CIDRα2.8 domain of PF3D7_0100100, for which the proportions of peptides recognized by Malian adult sera were significantly lower than those of the corresponding ATS domain. This finding may reflect the high diversity of CIDRα2.8 domains and result in a slower accumulation of seroreactivity. Indeed, CD36-binding PfEMP1s exhibit greater amino acid diversity than non-CD36-binding PfEMP1s (11). In contrast, CIDRα1 domains such as the domain present in PfDd2_040005100 are more conserved (35), which may explain similar rates of serologic recognition for the CIDRα1 and corresponding ATS region. This is consistent with a previous longitudinal study that found childhood acquisition of IgG antibodies occurring more rapidly for CIDRα1 domains than for CD36-binding domains such as CIDRα2.8 (37). This slower accumulation of antibody activity to the diverse CD36-binding PfEMP1 domains may shed light on why immunity to severe malaria appears to develop rapidly in early childhood, whereas susceptibility to uncomplicated malaria continues into adulthood.

Our results indicate that a significant increase in serologic response to malaria antigens may require multiple transmission seasons to develop. Malaria immunity is acquired gradually with age and malaria exposure (38), but the results of this study suggest that overall pediatric responses to the CIDRα1 domain did not significantly change over the course of one malaria transmission season. Across the variants analyzed, pediatric serologic responses increased for some peptides from the start of the transmission season to peak season. However, these responses largely waned from the peak to the end of the transmission season. Additionally, protein microarray data has shown that children mount increases in whole-protein seroreactivity after a clinical malaria infection, although this response appeared to wane to pre-season levels within the same transmission season (within 3–4 months) (39). This evidence illustrates the requirement for repeated exposure to malaria antigens to maintain natural immunity. Our results identified a small subset of peptides (6.65%) against which the pediatric cohort retained a significantly higher seroreactivity through the end of the transmission season. Future studies should utilize peptide microarrays to analyze serum samples from the same participants collected over multiple malaria transmission seasons to track serologic responses across multiple years, as it is possible that a clinically important change in serologic response only occurs after several malaria transmission seasons.

Our study had some limitations. The sample size in this study was limited to 10 Malian adults and 10 children, and a larger sample would increase the power to detect more subtle differences between groups. Our study indicates that the EPCR-binding region of a PfEMP1 may be an important site for the development of natural immunity to malaria. However, because of this limitation, large-scale follow-up studies are required to corroborate if our findings reflect patterns typically found in malaria-exposed populations, as well as identify the potential clinical importance of these antibody responses to the functional region of the CIDRα1 domain. Additionally, the peptide microarray used in this study includes only linear epitopes on short peptide segments, yet some B-cell epitopes are conformational or discontinuous and can only be detected when the antigen is in its three-dimensional conformation (40). However, most antibodies bind to short amino acid sequences (41), and the 16-amino acids length residues display some secondary structure, which could help detect some conformational epitopes. Our approach may thus have been unable to detect important epitopes, and techniques that can accommodate conformational epitopes may be useful in future studies to confirm the results observed here.

More work is needed to determine why peptides from EPCR-binding regions were more likely to be serorecognized, as it is possible that this is related to the three-dimensional protein structure of the CIDRα1, which contains a protruding hydrophobic patch that binds the hydrophobic groove of the EPCR (16). This exposure may allow for greater detection by the host immune system, whereas other amino acid residues may be comparatively hidden. Additional studies should be conducted to investigate whether antibodies against the binding region of the CIDRα1 domain can neutralize parasite binding to the EPCR, reducing virulence.

Future studies would ideally record peptide serologic responses immediately before and after a severe malaria episode to identify PfEMP1 peptides with an increased antibody response or an increase in the number of serorecognized epitopes after a severe episode to determine acute changes in serologic response, as well as continue monitoring for long term convalescent responses. However, it is challenging to identify children most at risk for severe malaria and obtain sera before an episode. Instead, broad pre-season pediatric population sampling for peptide serologic responses could indicate how serologic responses to the EPCR-binding peptide sequences of PfEMP1s differ between age groups and are associated with the subsequent development of severe and uncomplicated malaria. A future study could estimate the age when children reach a threshold level of serorecognition of PfEMP1 variants to identify the age at which children reach a threshold antibody response that predicts immunity to severe malaria. Tracking the extent of newly serorecognized epitopes for Malian children after each successive malaria transmission season could reveal approximately how many exposure cycles to PfEMP1 variants are required to reach such a threshold for serologic response.

Additional analysis of non-CD36 binding CIDRα1 variants or other PfEMP1 domains may also provide valuable insights. Analysis of multiple CD36-binding CIDRα2–6 variants may provide insight into the development of immunity to uncomplicated malaria. Evaluation of antibody accumulation patterns in other domains such as the DBLβ domains that bind ICAM-1 (42) could provide comparative insights into serologic responses within the CIDRα1 domain. Identifying the amount of serorecognition required to confer a threshold protective serologic response could be used to improve our understanding of the development of natural immunity to severe malaria and thereby inform vaccine development.

## SUPPLEMENTAL MATERIAL

Figure S1 msphere.00451-23-s0001.tif Multiple sequence alignment of CIDRa1 amino acid sequences.

Figure S2 msphere.00451-23-s0002.tif Seroreactivity to the CIDRα1 domain vs. CIDRα2.8 domain.

Figure S3 msphere.00451-23-s0003.tif Changes in pediatric seroreactivity for PfDd2_040005100.

Figure S4 msphere.00451-23-s0004.tif Changes in pediatric seroreactivity for PF3D7_0425800.

Figure S5 msphere.00451-23-s0005.tif Changes in pediatric seroreactivity for PF3D7_0533100.

Figure S6 msphere.00451-23-s0006.tif Changes in pediatric seroreactivity for PF3D7_0600200.

Figure S7 msphere.00451-23-s0007.tif Changes in pediatric seroreactivity for PF3D7_1150400.

Supplemental Legends msphere.00451-23-s0008.docx Legends for all supplemental tables and figures.

Table S1 msphere.00451-23-s0009.tif Participant demographic information.

Table S2 msphere.00451-23-s0010.tif Additional *P*-values for peptide recognition percentage among the non-binding vs. potential binding regions of PfEMP1 CIDRα1 domain variants.

ASM does not own the copyrights to Supplemental Material that may be linked to, or accessed through, an article. The authors have granted ASM a non-exclusive, world-wide license to publish the Supplemental Material files. Please contact the corresponding author directly for reuse.
