# Supplementary figures and images for: Natural immunity to malaria preferentially targets the endothelial protein C receptor-binding regions of PfEMP1s
Source: mSphere. 2023 Oct 4;8(5):e00451-23. doi: 10.1128/msphere.00451-23 (PMC10597466; doi:10.1128/msphere.00451-23)

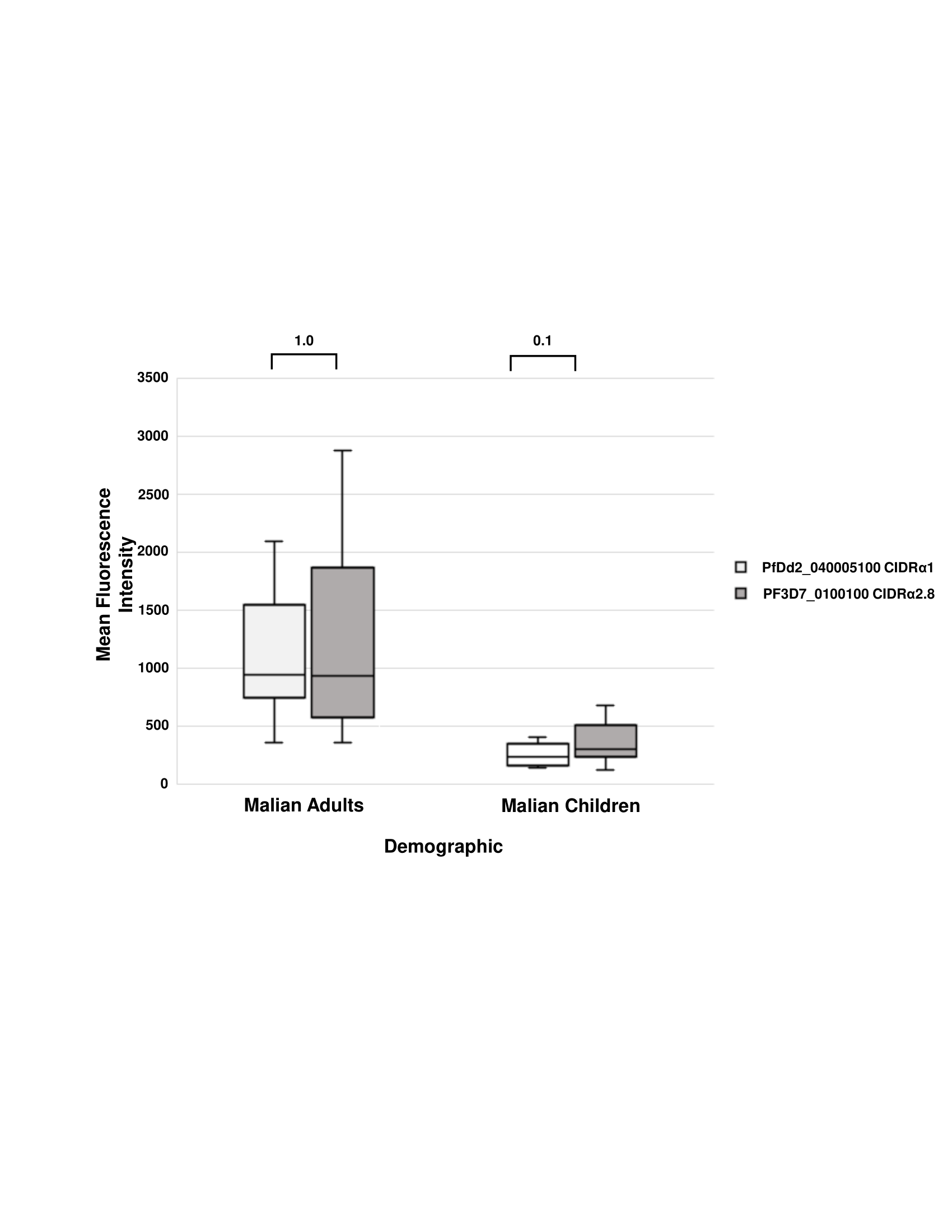

Supplement: Figure S2 — Seroreactivity to the CIDRα1 domain vs. CIDRα2.8 domain. [file msphere.00451-23-s0002.tif]

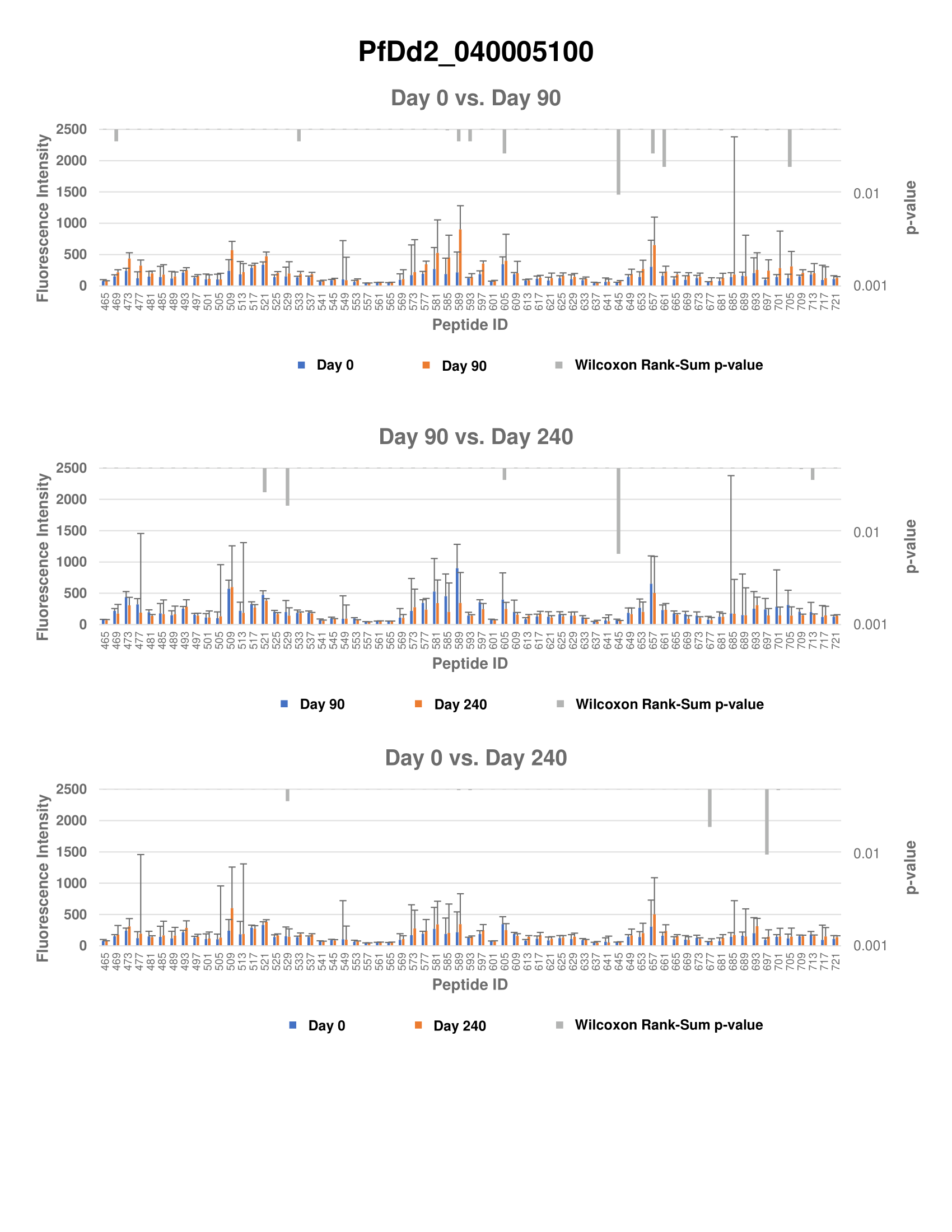

Supplement: Figure S3 — Changes in pediatric seroreactivity for PfDd2_040005100. [file msphere.00451-23-s0003.tif]

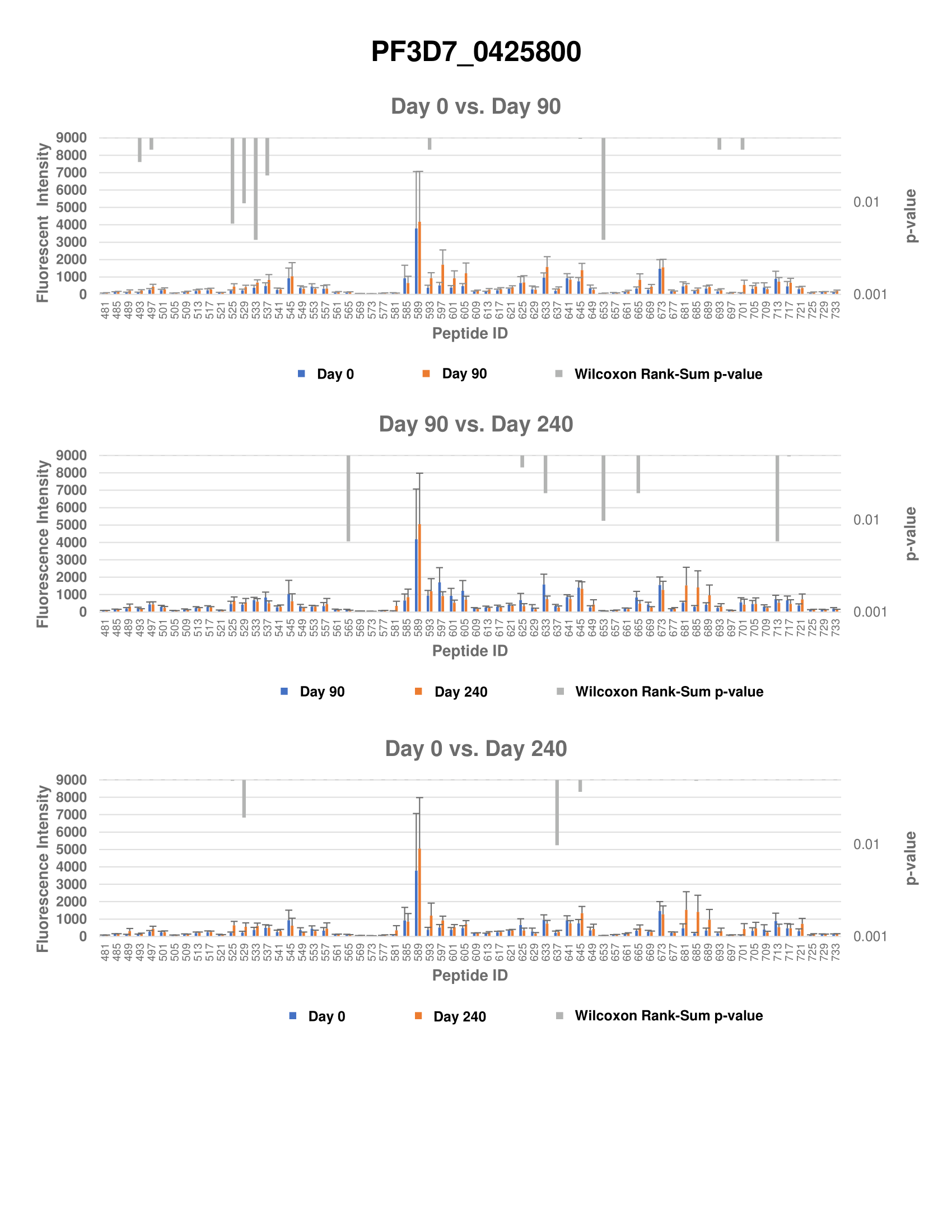

Supplement: Figure S4 — Changes in pediatric seroreactivity for PF3D7_0425800. [file msphere.00451-23-s0004.tif]

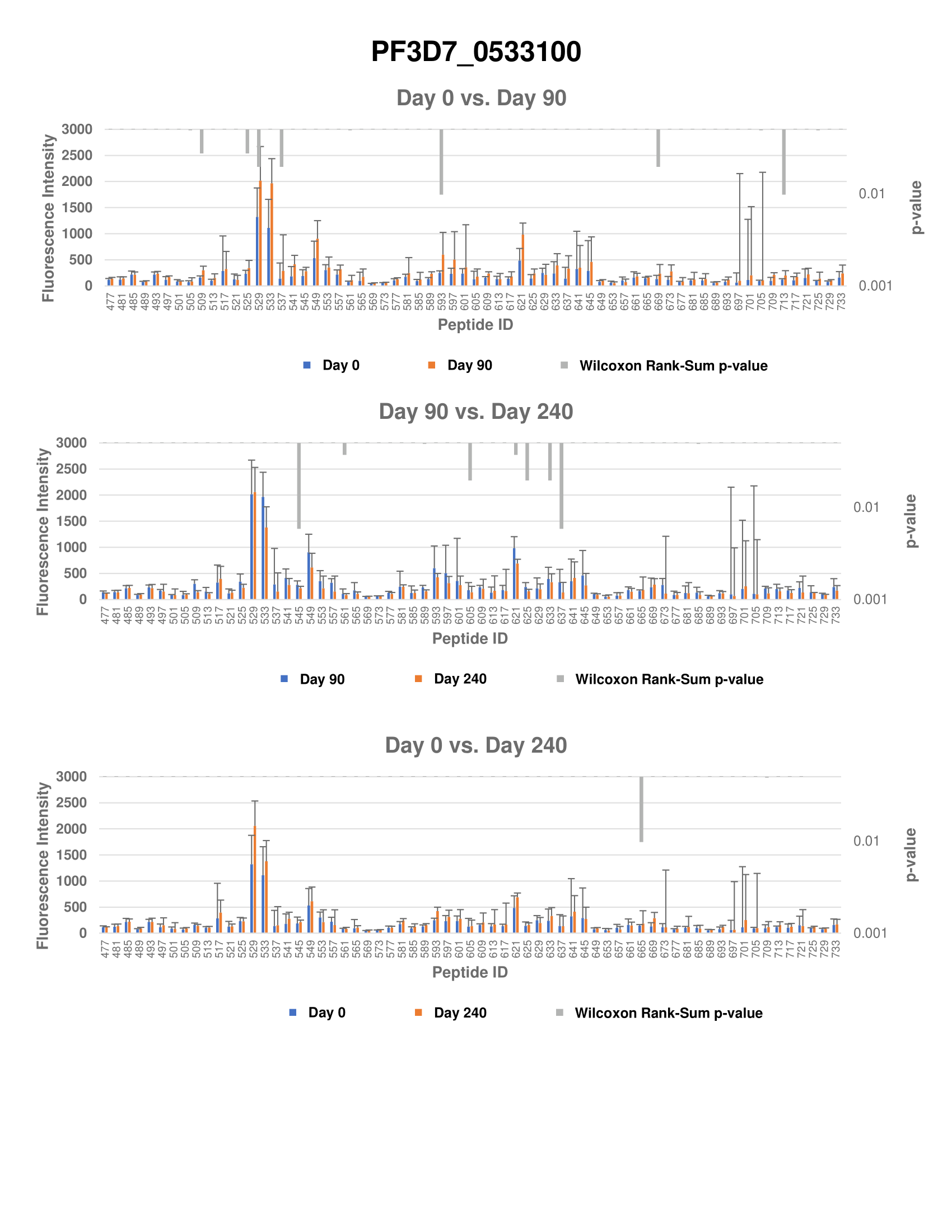

Supplement: Figure S5 — Changes in pediatric seroreactivity for PF3D7_0533100. [file msphere.00451-23-s0005.tif]

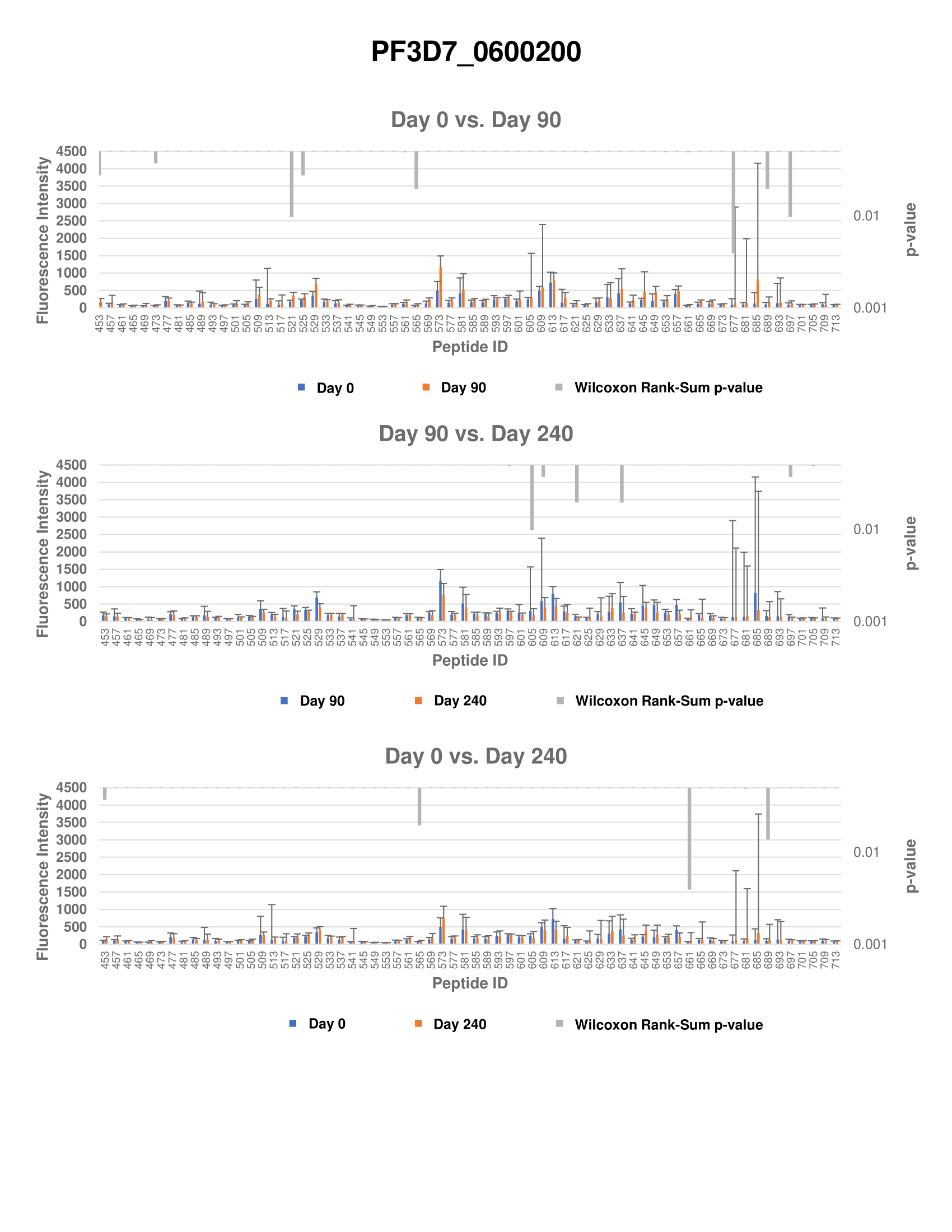

Supplement: Figure S6 — Changes in pediatric seroreactivity for PF3D7_0600200. [file msphere.00451-23-s0006.tif]

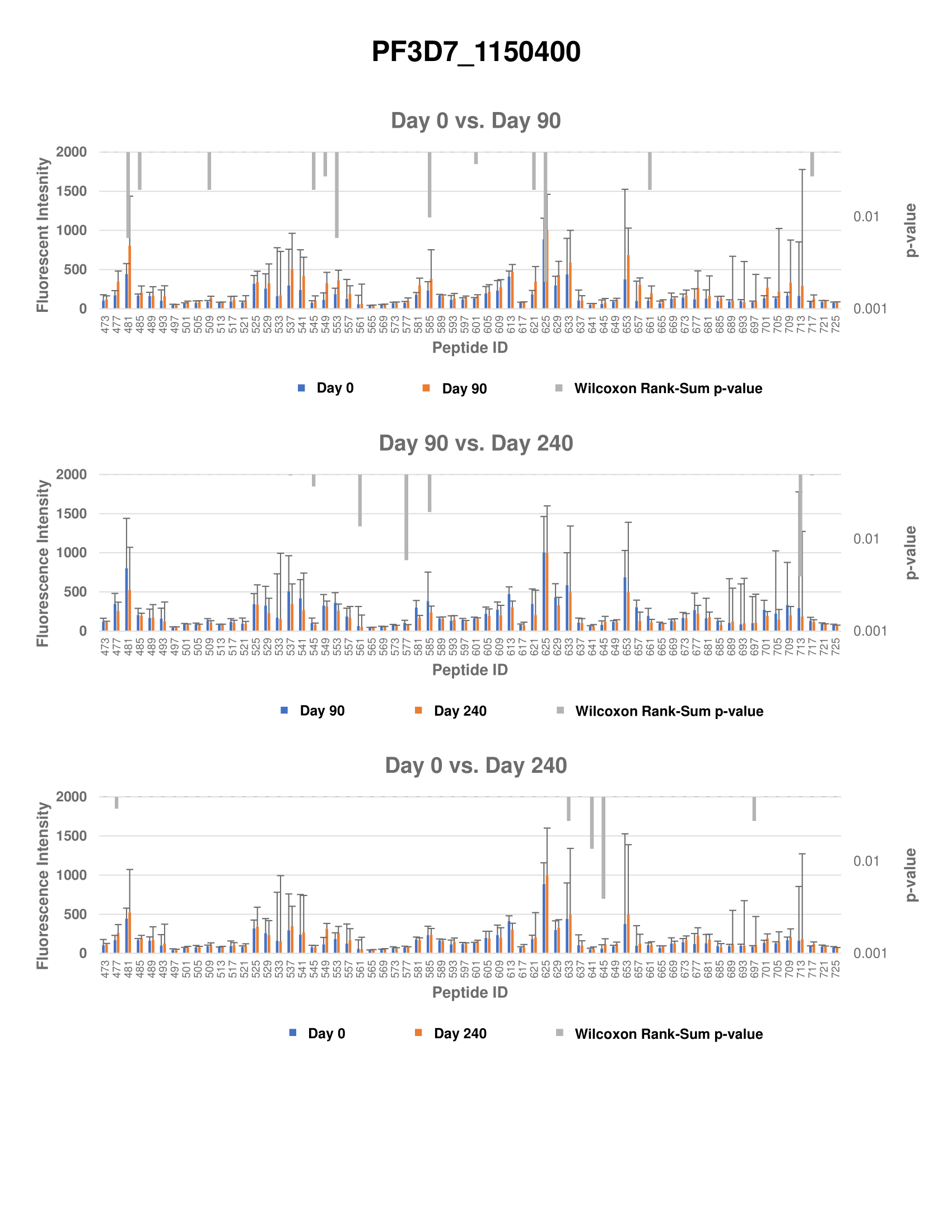

Supplement: Figure S7 — Changes in pediatric seroreactivity for PF3D7_1150400. [file msphere.00451-23-s0007.tif]

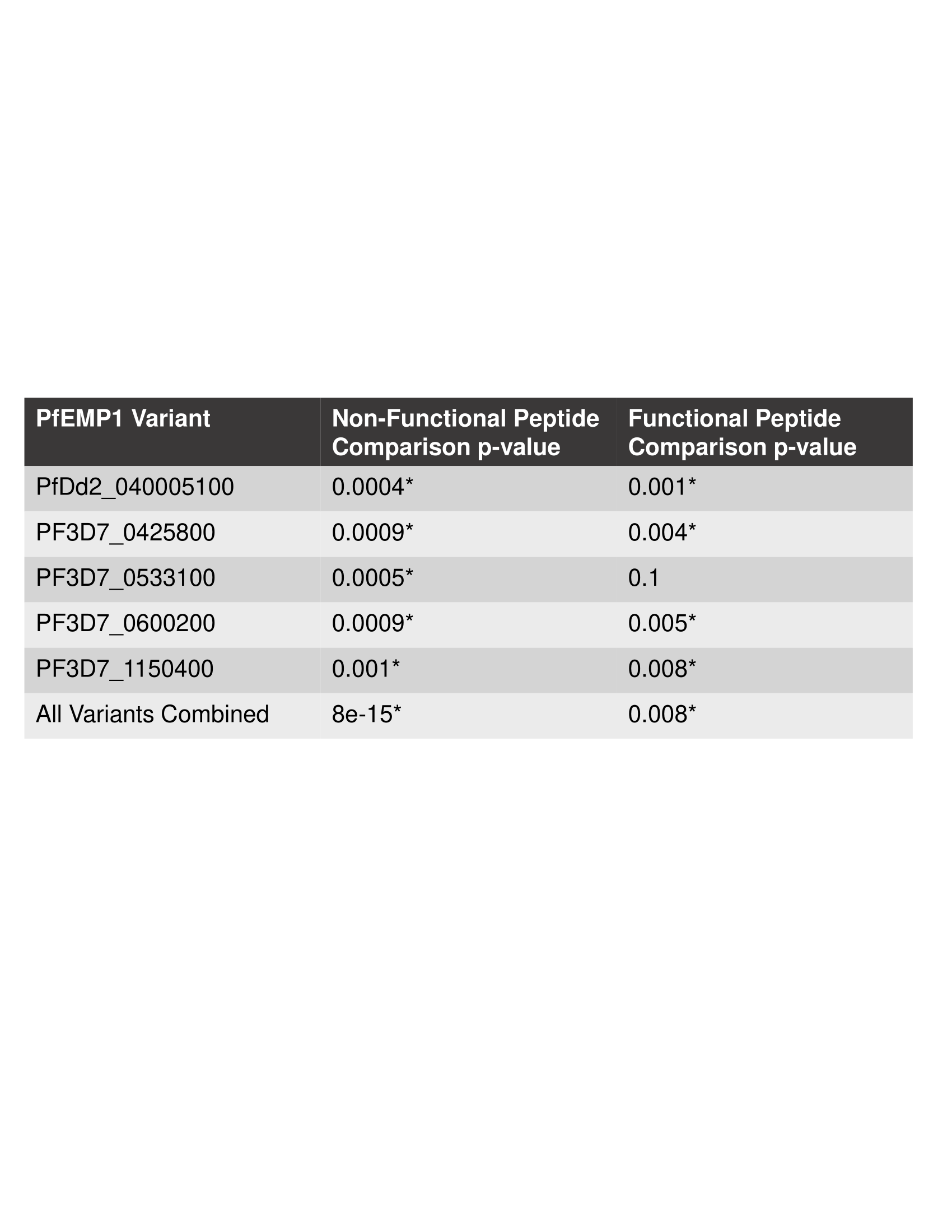

Supplement: Table S2 — Additional P-values for peptide recognition percentage among the non-binding vs. potential binding regions of PfEMP1 CIDRα1 domain variants. [file msphere.00451-23-s0010.tif]
