# Supplementary material for: Natural immunity to malaria preferentially targets the endothelial protein C receptor-binding regions of PfEMP1s
Source: mSphere. 2023 Oct 4;8(5):e00451-23. doi: 10.1128/msphere.00451-23 (PMC10597466; doi:10.1128/msphere.00451-23)
Supplement: Supplemental Legends — Legends for all supplemental tables and figures. [file msphere.00451-23-s0008.docx]

Table S1: **Participant Demographic Information.** Sex, age, and malaria incidence information for Malian adult (n=10), Malian child (n=10), and North American adult participants (n=5).

Table S2: **Additional p-values for peptide recognition percentage among the non-binding vs. potential binding regions of PfEMP1 CIDRα1 domain variants.** The table depicts the p-values that accompany the red triangles in Fig. 4, which compare each set of adult peptides to the corresponding pediatric peptides. An asterisk (*) indicates a value significant at α= 0.05. All significant values indicate that Malian adult serorecognition proportions were significantly higher than corresponding pediatric proportions.

Fig. S1: **Multiple sequence alignment of CIDRa1 amino acid sequences.** The alignment was generated using the MUSCLE algorithm. The color of each position indicates its level of conservation, with darker colors representing more conserved positions. The sequence logo underneath shows the prevailing amino acid at each position and the consensus sequence.

Fig. S2: **Seroreactivity to the CIDRα1 domain vs. CIDRα2.8 domain.** Average fluorescence intensity for each adult (N=10) and pediatric (N=10) participant in the CIDRα1 domain of PfDd2_040005100 and CIDRα2.8 domain of PF3D7_0100100 were compared using a paired Wilcoxon Signed Rank test (α = 0.05). Each box plot shows the median, bracketed by the lower 25th and upper 75th percentiles, and the minimum and maximum values.

Fig. S3: **Changes in pediatric seroreactivity for PfDd2_040005100.** Median fluorescence intensity in the CIDRα1 domain for Malian pediatric sera (n=10) was collected at three time points: the beginning of the malaria transmission season (Day 0), the peak of transmission season (Day 90), and the end of the transmission season (Day 240). Timepoints were compared using a paired Wilcoxon Rank Sum test to identify peptides against which antibody response significantly changes, indicated with a grey p-value bar. (A) Changes from Day 0 to Day 90 (B) Changes from Day 90 to Day 240 (C) Changes from Day 0 to Day 240

Fig. S4: **Changes in pediatric seroreactivity for PF3D7_0425800.** Median fluorescence intensity in the CIDRα1 domain for Malian pediatric sera (n=10) was collected at three time points: the beginning of the malaria transmission season (Day 0), the peak of transmission season (Day 90), and the end of the transmission season (Day 240). Timepoints were compared using a paired Wilcoxon Rank Sum test to identify peptides against which antibody response significantly changes, indicated with a grey p-value bar. (A) Changes from Day 0 to Day 90 (B) Changes from Day 90 to Day 240 (C) Changes from Day 0 to Day 240

Fig. S5: **Changes in pediatric seroreactivity for PF3D7_0533100.** Median fluorescence intensity in the CIDRα1 domain for Malian pediatric sera (n=10) was collected at three time points: the beginning of the malaria transmission season (Day 0), the peak of transmission season (Day 90), and the end of the transmission season (Day 240). Timepoints were compared using a paired Wilcoxon Rank Sum test to identify peptides against which antibody response significantly changes, indicated with a grey p-value bar. (A) Changes from Day 0 to Day 90 (B) Changes from Day 90 to Day 240 (C) Changes from Day 0 to Day 240

Fig. S6: **Changes in pediatric seroreactivity for PF3D7_0600200.** Median fluorescence intensity in the CIDRα1 domain for Malian pediatric sera (n=10) was collected at three time points: the beginning of the malaria transmission season (Day 0), the peak of transmission season (Day 90), and the end of the transmission season (Day 240). Timepoints were compared using a paired Wilcoxon Rank Sum test to identify peptides against which antibody response significantly changes, indicated with a grey p-value bar. (A) Changes from Day 0 to Day 90 (B) Changes from Day 90 to Day 240 (C) Changes from Day 0 to Day 240

Fig. S7: **Changes in pediatric seroreactivity for PF3D7_1150400.** Median fluorescence intensity in the CIDRα1 domain for Malian pediatric sera (n=10) was collected at three time points: the beginning of the malaria transmission season (Day 0), the peak of transmission season (Day 90), and the end of the transmission season (Day 240). Timepoints were compared using a paired Wilcoxon Rank Sum test to identify peptides against which antibody response significantly changes, indicated with a grey p-value bar. (A) Changes from Day 0 to Day 90 (B) Changes from Day 90 to Day 240 (C) Changes from Day 0 to Day 240
